# Consensus on the Clinical Approach to Moderate-to-Severe Atopic Dermatitis in Spain: A Delphi Survey

**DOI:** 10.1155/2020/1524293

**Published:** 2020-04-07

**Authors:** Joaquín Sastre, Esther Serra Baldrich, José Carlos Armario Hita, L. Herráez, Ignacio Jáuregui, Ana Martín-Santiago, Javier Ortiz de Frutos, Juan Francisco Silvestre, Antonio Valero

**Affiliations:** ^1^Service of Allergy, Fundación Jiménez Díaz, Madrid, CIBERES, Instituto Carlos III, Department of Medicine, Universidad Autónoma de Madrid, Madrid, Spain; ^2^Dermatology Unit, Hospital de la Santa Creu i Sant Pau, Barcelona, Spain; ^3^Service of Dermatology, Hospital Universitario Puerto Real, University of Cádiz, Cádiz, Spain; ^4^Service of Allergy, Hospital Universitario 12 de Octubre, Madrid, Spain; ^5^Service of Allergy, Hospital Universitario Cruces, Barakaldo, Vizcaya, Spain; ^6^Service of Dermatology, Hospital Universitari Son Espases, Palma, Spain; ^7^Service of Dermatology, Hospital Universitario 12 de Octubre, Madrid, Spain; ^8^Service of Dermatology, Hospital General Universitario de Alicante, Alicante, Spain; ^9^Section of Allergy, Hospital Clinic i Provincial de Barcelona, IDIBAPS, Universitat de Barcelona, Barcelona, Spain

## Abstract

**Background:**

The purpose of this study was to gather information on the current assessment and management of patients with moderate-to-severe AD in routine daily practice.

**Methods:**

A cross-sectional two-round Delphi survey with the participation of dermatologists and allergologists throughout Spain was conducted. They completed a 46-item questionnaire, and consensus was defined when responses of ≥80% of participants coincided in the categories of a 5-point Likert scale for that item.

**Results:**

A total of 105 specialists (aged 40–59 years) completed the two rounds. Participants agreed regarding the consideration of AD as a multifaceted disease and the differences in clinical presentation of AD according to the patient's age. It is recommendable to perform a skin biopsy to exclude early stage T-cell cutaneous lymphoma, psoriasis, or dermatitis herpetiformis, among others (99.1%). Also, consensus was reached regarding the use of the SCORAD index to quantify the severity of the disease (86.7%), the use of wet wraps to increase the effect of topical corticosteroids (90.4%), the usefulness of proactive treatment during follow-up (85.6%) and tacrolimus ointment (91.2%) to reduce new flares, and the fact that crisaborole is not the treatment of choice for severe AD (92.4%). AD was not considered a contraindication for immunotherapy in patients with allergic respiratory diseases (92.4%). In patients with severe AD, the use of immune response modifier drugs (97.6%) or phototherapy (92.8%) does not sufficiently cover their treatment needs. Consensus was also obtained regarding the role of the new biologic drugs (93.6%) targeting cytokines involved in the Th2 inflammatory pathway (92.0%) and the potential role of dupilumab as first-line treatment (90.4%) in moderate-to-severe AD patients.

**Conclusion:**

This study contributes a reference framework to the care of AD patients. There is no diagnostic test or biomarkers to direct treatment or to assess the severity of the disease, and many therapeutic challenges remain.

## 1. Introduction

Atopic dermatitis (AD) is a chronic, pruritic, relapsing inflammatory skin condition, commonly affecting children and, to a lesser extent, adults. Infants with AD may develop a typical progression of atopic disorders, including allergic rhinitis, food allergy, and asthma at certain ages, a sequence commonly referred to as the atopic march [1, 2]. AD diagnoses are continuously on the rise, oscillating between 10% and 20% of the pediatric population. The disease affects 1–10% of adults worldwide, and recent studies have suggested that adult AD is more common than previously thought [3, 4]. Although AD is not a life-threatening condition, it poses a significant social, psychological, and economic burden [5]. The negative psychosocial impact of AD on quality of life is well established [5, 6], with itching, scratching, sleep loss, and social embarrassment being among the most commonly reported difficulties contributing to school, work, and social struggles. Depression and anxiety have been reported to be also more common in adults with AD, with these psychiatric symptoms being influenced by AD disease severity and the degree of impairment of quality of life [6].

The presentation of AD depends on age and ranges from papulovesicles to lichenified plaques. The pathogenesis of AD is multifactorial, including genetic, and environmental factors. Also, AD is characterized by skin barrier defects, immunologic dysfunction, and alterations in the skin microbiome [7–9]. However, a number of cytokines and mediators involved in Th2, Th22, Th17, and Th1 pathways appear to be important in AD pathogenesis, and there is currently an increasing interest in developing targeted therapies, especially for patients with moderate-to-severe forms of the disease not responding to conventional treatments [10–12]. Also, the disease is frequently associated with other comorbid skin conditions and extracutaneous diseases [13, 14]. The causative mechanisms underlying these associations are poorly understood, but treating physicians should be aware of these associations while seeking to alleviate the burden for patients with AD [15].

Available evidence on different aspects of the disease is uncertain and scarce at the population level. There are two studies on the prevalence of severe AD in adults in Spain, one in three different areas (Asturias, Catalonia, and the Balearic Islands) in 2015-2016 and the other one in Madrid in 2016-2017; the estimated prevalence was 0.08% and 0.028% (0.015% for children), respectively [16, 17]. AD was considered severe when the patient received a systemic immunosuppressant or a biologic (omalizumab, rituximab) during follow-up or had been hospitalized because of AD [16]. However, presently, there exists no gold standard for evaluating the severity of AD [18].

A study based on data from the 2016 National Health and Wellness Survey conducted in France, Germany, Italy, Spain, and the United Kingdom revealed that AD is associated with a significant disease burden and higher prevalence of atopic and psychological comorbidities, impaired health-related quality of life, lower work productivity, increased activity impairment, and increased healthcare utilization [19]. The Spanish Society of Allergology and Clinical Immunology carried out two wide-scale studies regarding the epidemiologic, clinical, and socioeconomic factors of the main allergic disorders in Spain, including AD [20, 21].

However, no previous studies have evaluated the clinical approach to patients with AD at a national level in Spain. Therefore, a cross-sectional Delphi survey was designed to gather information on the assessment and management of patients with moderate-to-severe AD in conditions of routine clinical practice. The results of this study will be helpful to identify some of the unmet medical needs currently present in the care of these patients.

## 2. Methods

### 2.1. Study Design

A cross-sectional two-round Delphi survey was conducted over a 5-month period (between July 13 and December 5, 2017) with the participation of dermatologists and allergologists throughout Spain. The objective of the study was to develop a consensus document on the clinical approach to moderate-to-severe AD patients. The aims of the consensus were as follows: (a) to help clinicians in the assessment and management of AD patients in daily practice, (b) to know the current status of the care of patients with AD in Spain, and (c) to identify potential areas to improve outcomes, including early recognition of hallmark signs and symptoms to ensure correct diagnosis and use of evidence-based tools to determine disease impact and guide treatment decisions, and to promote patient engagement and adherence to therapies.

The Delphi method is generally accepted as a powerful means of reaching consensus and generating ideas among responders on a number of issues related to health problems in conditions of low grade evidence, knowledge, or application [22]. Briefly, the method involves sending a questionnaire to the responders and analyzing their response. This is then used to develop a new questionnaire and the cycle is repeated. Three methodological aspects are important in a Delphi study. First, responders or panelists are not aware of the identity of the other responders, to ensure that their responses are independent. Second, participants respond individually to avoid group domination by certain individuals. Third, mathematical voting procedures are used to permit the ranking of items. Likewise, there are no set guidelines for deciding on the optimum number of Delphi participants as this is likely to change depending on the purpose of the Delphi survey. The original Delphi method involves three or more rounds, whereas the modified technique is limited to two rounds to avoid losses of acceptable response rates due to prolonged duration of the process and the negative influence on the interest of the panelists. In the present study, a modified Delphi method was used to reach consensus.

### 2.2. Participants and Procedures

At the beginning of the project, a scientific committee formed by nine specialists, five dermatologists, and four allergologists with proven experience and interest in AD was established. Participants were authors of relevant research publications and were renowned professionals in the care of allergic patients, with expertise in AD. Two study coordinators (JS, ES) supervised the progression of the study, including recruitment of participants and results of analysis of data, as well as other organizational and logistic aspects of the project.

Items to be included in the study questionnaire were selected by members of the scientific committee based on a search of the literature to identify previously conducted studies with high level of evidence, such as systematic reviews and meta-analyses, and key primary studies focused on the field of moderate-to-severe AD in adult patients. Candidates to participate in the study were proposed by the study coordinators and members of the scientific committee. They were specialists in dermatology or allergology involved in the care of patients with AD in public hospitals and some private consultations throughout Spain, preferably with 5 or more years of experience and attending at least 10 patients with AD per month. The study questionnaire was lodged in an Internet microsite to which specialists who agreed to participate accessed via a weblink with the user's password.

The initial draft included 47 items, two of which were removed after the first round and a new item was added. Therefore, the final questionnaire was composed of a set of 46 items, in which questions were grouped into seven sections: definition and diagnosis (14 items), differential diagnosis (1 item), severity of AD (2 items), etiology and physiopathology (4 items), comorbidities of AD (4 items), health-related quality of life (2 items), and treatment and follow-up (19 items). Questions were formulated so that they could be answered using a 5-point Likert scale: 1: “strongly disagree,” 2: “disagree,” 3: “neither agree nor disagree,” 4: “agree,” and 5: “strongly agree.” The study questionnaire is described in the Supplementary Materials.

### 2.3. Statistical Analysis

Only full completed questionnaires were considered. The consensus criteria included “unanimity” when 100% of the participants agreed on the same category of the Likert scale, “consensus” when agreement involved ≥80% of the participants, “majority” when agreement involved ≥70% of participants, and “discrepancy” when agreement involved <70% of the participants. For the purpose of the analysis, “unanimity” and “consensus” groups were considered together as a consensus. Descriptive statistics included frequencies and percentages. The analysis was carried out trough consultation and analysis of the answers in the database from the online platform.

## 3. Results

### 3.1. Characteristics of Participants

Of a total of 200 dermatologists and allergologists who were invited to participate in the study, 125 (62.5%) accepted and completed the first Delphi round, 105 (84%) of which completed the second round. The final participation rate was 52.5%. Of the 105 participants, 59 (56.2%) were dermatologists and 46 (43.8%) allergologists. The general profile of participants included a mean age between 40 and 59 years, 5 or more than 5 years of experience in the care of patients with AD with at least 10 patients with AD visiting per month (both children and adults), and 46.7% working in public hospitals, with all of them having available biologic drugs in the workplace.

### 3.2. General Results

After the two Delphi rounds, consensus was reached on 36 of the 46 items (78.3%). There were 3 items where the agreement involved ≥70% of the participants (6.5%) and there was discrepancy in 7 items (15.2%). As shown in Table 1, the percentages of consensus ranged between 50% and 100%, with the lowest percentages being in the sections of severity of AD and comorbidities and the highest percentages in the sections of differential diagnosis, etiology and physiopathology, and quality of life.

### 3.3. Definition and Diagnosis of AD

Of the 14 items included in this section, 12 (85.7%) achieved consensus, and there was discrepancy in the remaining 2 (14.3%) (Table 2). Participants agreed regarding the consideration of AD as a multifaceted disease derived from the interaction of multiple factors, including the skin components (96.8%), the immune system (97.6%), skin microbiome (89.6%), and genetic (98.4%) and environmental (98.4%) factors. AD is diagnosed clinically (97.6%) using the criteria of Hanifin and Rajka (94.3%), with the clinical presentation depending on the patient's age (92.8%), and currently lacking validated biomarkers that can help in the diagnosis of AD (83.2%). There were discrepancies in the usefulness of the classification of intrinsic and extrinsic AD as the basis of specific avoidance strategies (60%) and eosinophil count as a biomarker of AD (37.1%).

### 3.4. Differential Diagnosis

Participants agreed on the single item of this section, i.e., performing a skin biopsy to exclude other conditions, such as early stage T-cell cutaneous lymphoma, psoriasis, or dermatitis herpetiformis (99.1%) (Table 3).

### 3.5. Severity of AD

Of the two items, consensus was reached on one regarding the use of the SCORAD (scoring atopic dermatitis) index to quantify the severity of the disease (86.7%). There was discrepancy about eczema area and severity index (EASI) score as a validated scale that is not used in routine clinical practice (58.1%) (Table 3).

### 3.6. Etiology and Physiopathology

Of the four items, consensus was reached in all of them with the highest percentages regarding that “currently there is an unmet need in the treatment of moderate-severe AD” (98.4%) and the statement that “a high percentage of patients with AD also present food allergy, allergic rhinitis, or asthma” (94.4%) (Table 3).

### 3.7. Comorbidities

Of the four items, two achieved consensus regarding the fact that DA in children is frequently associated with allergic comorbidities (91.4%) and that children with AD are more prone to suffer from mental disorders (84.8%). There was no consensus about the association of AD in adults with allergic disorders (78.1%) and a higher relative risk for immune-mediated inflammatory diseases (43.8%) (Table 3).

### 3.8. Health-Related Quality of Life

Consensus was reached in the two items regarding AD as a cause of marked psychological anxiety (91.2%) and the usefulness of patient-oriented SCORAD (PO-SCORAD) for the self-assessment of AD in children, integrating the perspectives of the physician and the patient in the management of the disease (91.2%) (Table 3).

### 3.9. Treatment and Follow-Up

Of the 19 items regarding different aspects of treatment and follow-up of patients with AD, 14 (73.7%) obtained consensus, whereas most participants agreed on 2 items (10.5%) and there were discrepancies in 3 (15.8) (Table 4). In the presence of positive prick tests, participants agreed on avoidance of suspected allergens (83.8%) and dietary foods (91.4%). There was consensus on the use of wet wraps to increase the effect of topical corticosteroids (90.4%), the usefulness of proactive treatment during follow-up (85.6%) and tacrolimus ointment (91.2%) to reduce new flares, and the fact that crisaborole is not the treatment of choice for severe AD (92.4%). Most of the participants (72.4%) believed that the simultaneous combination on the same location of topical glucocorticoids and topical calcineurin inhibitors is not useful. Also, there was consensus that there are no enough bibliographic references supporting the use of antihistamines to alleviate pruritus (85.7%) but there was discrepancy regarding the usefulness of antihistamines as systemic treatment of AD (64.8%). Most participants agreed on the positive effects of allergen-specific immunotherapy (SIT) in some sensitized patients (76.2%) and reached consensus that the item of AD is not a contraindication for immunotherapy in patients with allergic respiratory diseases (92.4%). In patients with severe AD, the use of immune response modifier drugs (97.6%) or phototherapy (92.8%) does not sufficiently cover their treatment needs. Participants agreed on the adequate risk-benefit ratio of cyclosporine (80.0%) but disagreed on the adequate risk-benefit ratio of phototherapy (69.5%). Finally, there was consensus on the role of the new biologic drugs (93.6%) targeting cytokines involved in the Th2 inflammatory pathway (92.0%) and the potential role of dupilumab as first-line treatment (90.4%) in moderate-to-severe AD patients. By contrast, there was discrepancy regarding the position of Janus kinase (JAK) inhibitors in the treatment of AD (60.0%).

## 4. Discussion

The aim of the Delphi method is to obtain an opinion, level of agreement, or consensus on a current topic or concern from among a group of specialists or experts. This is an iterative and anonymous process with controlled feedback and analysis of the results widely used in health sciences. For this study, the percentage of unanimity, consensus, majority, and discrepancy has been decided taking into account the standard percentages that are usually used for a Delphi questionnaire of these characteristics and by agreement of the entire scientific committee. Although the Delphi methodology has been applied in various dermatological diseases [23–31], no previous studies comprising a consensus of opinion have been published on AD using the Delphi technique. In a multicenter international project, a Delphi study was conducted to reach consensus between different stakeholders on a core set of domains and items for registries of atopic eczema patients to collect data for research focused on photo- and systemic immunomodulatory therapies [32]. In this respect, given the paucity of Delphi studies on AD, the present survey fills a gap in the literature.

With respect to the percentages of panelists having completed the rounds of questions set, the data varies depending on the characteristics of the study, including the number of experts, survey distribution methods, number of rounds, and face-to-face meetings. The overall participation rate in our study was 52.5%, but the percentage of participants having completed the two Delphi rounds was 84%, being in the upper band of the range over 60–80% reported in the literature [33, 34]. In addition, the total number of 46 items was adequate, as high number of items is associated with significantly lower response rates [35].

In the opinion of the panelists, consensus was obtained regarding the consideration of AD as a multifaceted disease caused by the interaction of multiple factors. Recent studies of the etiopathogenesis of AD have highlighted the complex interplay among skin barrier deficiency, immunological derangement, which contributes to the development, progression, and chronicity of the disease [36]. Abnormalities in filaggrin, another stratum corneum constituent, and tight junctions induce and/or promote skin inflammation. This inflammation, in turn, can further deteriorate the barrier function by downregulating a myriad of essential barrier-maintaining molecules [36]. The panel also agreed that the diagnosis of AD remains clinical without a reliable biomarker to confirm the diagnosis or assess the severity of the disease. Currently, biomarkers cannot be applied in daily practice for diagnosing AD, although they will play an increasingly important role in AD research and personalized medicine [37]. There were discrepancies regarding the usefulness of the classification of intrinsic and extrinsic AD as the basis of specific avoidance strategies, eosinophil count as a biomarker of AD, and the use of EASI in clinical practice.

There was also consensus about differences in the clinical presentation of AD according to the patient's age. Even though many common features exist, there are significant differences between the clinical characteristics of children, adolescents, and adult AD subgroups [38]. Moreover, the lack of standardization of outcome measures for clinical signs of AD, such as clinical signs measured with a physician-assessed instrument and symptoms measured with a patient-assessed instrument or control of flares, hampers comparison among studies [39]. Participants also agreed on the need to perform a skin biopsy to exclude other conditions, including early stage T-cell cutaneous lymphoma, psoriasis, or dermatitis herpetiformis. In this respect, the clinical characteristics and the hallmark of Sézary syndrome, a leukemia variant of T-cell cutaneous lymphoma, often share striking similarities with AD [40].

In relation to comorbid conditions, allergic diseases in adults and depression, anxiety, and behavioral disorders in children were items for which consensus was obtained. A recent meta-analysis of 35 studies found that children and adolescents with AD had significantly higher risk of total mental disorders than those without AD (odds ratio [OR] 1.65; 95% confidence interval [CI] 1.46–1.86), with an absolute risk of 12.6% [41]. Also, AD was recognized as the cause of considerable psychological anxiety. In fact, psychological stress is a significant contributor to AD disease course through its direct and indirect effects on immune response, cutaneous neuropeptide expression, and skin barrier function [42].

Consensus regarding treatment included the use of wet wraps to enhance the effect of topical corticosteroids, the use of proactive treatment in the affected area to reduce new flares, the recognition that crisaborole is not the treatment of choice for severe AD, the adequate benefit-risk ratio of cyclosporine in severe AD, the role of biologic drugs, targeting cytokines involved in allergic inflammation caused by Th2 helper lymphocytes, and the potential of dupilumab to become the new first-line treatment in patients with moderate-to-severe AD [43, 44]. A consensus was also reached on how to interpret skin prick test results. There was discrepancy in relation to the statement that first- and second-generation antihistamines are generally not useful in the systemic treatment of AD. Short-term, intermittent use of sedating antihistamines may be beneficial in the setting of sleep loss secondary to itch [45] but they reduce rapid eye movement (REM) sleep, produce daytime somnolence, impair learning, and reduce work efficiency [46]. It has been postulated that second-generation antihistamines may have anti-inflammatory effects. In human keratinocytes, histamine decreases the formulation of tight junctions and the expression of filaggrin, a gene responsible for AD, via histamine H1 receptor (H1R) [47]. Furthermore, histamine regulates IL-31, a cytokine involving the skin barrier and pruritus, through H1R and induces the production of thymus and activation-regulated chemokine (TARC) via H4R [47]. Recently, in a randomized, double-blind, placebo-controlled, parallel-group study, a novel selective H4R antagonist, ZPL-3893787, showed significant reductions of EASI and SCORAD scores as compared with placebo, but a nonsignificant difference in the reduction of pruritus [48].

The results of the present study should be interpreted taking into account the fact that responses to the questionnaire reflect what the specialists would do in the different scenarios posed by each question, which may differ slightly from their clinical practice. However, the Delphi method allowed exploring systematically different clinical, diagnostic, and treatment aspects of patients with AD, based on the qualified opinion of dermatology and allergology experts in the field. There is no diagnostic test or biomarkers to direct treatment or to assess the severity of the disease, and many therapeutic challenges remain. The extent to which activation of immune pathways in addition to Th2 signaling contributes to AD pathogenesis remains unknown [49], as well as whether new medications directed against targets believed to lead to AD will prove to be effective without significant risk.

In summary, this was a Delphi survey study of a sample of dermatology and allergology experts in the care of patients with AD. The present consensus document contributes as a reference framework for the care of adult patients with moderate-to-severe AD in clinical practice.

## Figures and Tables

**Table 1:** General results obtained for the different sections of the questionnaire.

Section	Items *n*	Consensus *n* (%)	Majority *n* (%)	Discrepancy *n* (%)
1. Definition and diagnosis	14	12 (85.7)	0	2 (14.3)
2. Differential diagnosis	1	1 (100)	0	0
3. Severity of AD	2	1 (50)	0	1 (50)
4. Etiology and physiopathology	4	4 (100)	0	0
5. Comorbidities of AD	4	2 (50)	1 (25)	1 (25)
6. Health-related quality of life	2	2 (100)	0	0
7. Treatment and follow-up	19	14 (73.7)	2 (10.5)	3 (15.8)

AD: atopic dermatitis.

**Table 2:** Results regarding definition and diagnosis of AD.

Item	Consensus (%)	Discrepancy (%)
(i) AD can also develop *de novo* in adults or young adults, or even in advanced age	93.6	
(ii) AD is a multifaceted disease that is derived from the interactions of multiple factors, including the skin components (cellular and extracellular components that form the skin barrier)	96.8	
(iii) AD is a multifaceted disease that is derived from the interactions of multiple factors, including the immune system (innate and adaptative)	97.6	
(iv) AD is a multifaceted disease that is derived from the interactions of multiple factors, including the skin microbiome	89.6	
(v) AD is a multifaceted disease that is derived from the interactions of multiple factors, including genetic factors	98.4	
(vi) AD is a multifaceted disease that is derived from the interactions of multiple factors, including environmental factors	98.4	
(vii) Currently, the diagnosis and assessment of the severity of AD are made on clinical grounds	97.6	
(viii) The clinical criteria defined by Hanifin and Rajka are used for the clinical diagnosis of AD	94.3	
(ix) The clinical presentation of AD depends on the age of the patients	92.8	
(x) With greater frequency, children < 2 years of age and adults present involvement of the face and neck; in addition, adults also present involvement of the flexor and extensor surfaces	94.3	
(xi) Some forms of presentation seen in adults include dermatitis of the head and neck, chronic eczema of the hands, and multiple zones of lichenification or prurigo	96.8	
(xii) The classification of “intrinsic” AD (not associated with IgE) and “extrinsic” AD (associated with IgE) has practical implications related to specific avoidance strategies in the management of the disease		60.0
(xiii) The blood eosinophil count is not a useful biomarker in AD		37.1^*∗*^
(xiv) Currently there are no validated biomarkers that help in the diagnosis of AD	83.2	

AD: atopic dermatitis; IgE: immunoglobulin E. ^*∗*^37.1% of disagreement.

**Table 3:** Results regarding differential diagnosis, severity of AD, etiology and physiopathology, comorbidities, and health-related quality of life.

Item	Consensus (%)	Majority (%)	Discrepancy (%)
Differential diagnosis			
(i) In certain situations, skin biopsy should be considered to exclude other conditions, such as early stage T-cell cutaneous lymphoma, psoriasis, or dermatitis herpetiformis	99.1		
Severity of AD			

(i) The SCORAD index is used to quantify the severity of the disease in order to assess the comparative efficacy of treatments and progression of the disease in routine clinical practice	86.7		
(ii) The EASI score is a validated scale that is not used in routine clinical practice			58.1

Etiology and physiopathology			
(i) A high percentage of patients with AD also present food allergy, allergic rhinitis, or asthma	94.4		
(ii) Treatments oriented to increase filaggrin expression can be useful in the management of AD in a particular group of patients	93.3		
(iii)Currently there is an unmet need in the treatment of moderate-severe AD	98.4		
(iv) Biologic drugs are especially promising for adult patients with moderate or severe forms of the disease	89.6		

Comorbidities of AD			
(i) AD in adults is frequently associated with allergic comorbidities		78.1	
(ii) AD in children is frequently associated with allergic comorbidities	91.4		
(iii) The relative risk of suffering from immune-mediated inflammatory diseases such as rheumatoid arthritis and chronic inflammatory bowel disease is higher in patients with AD than in the general population			43.8^*∗*^
(iv) Children with AD are more prone to suffer from mental disorders such as depression, anxiety, and behavior disorders	84.8		

Health-related quality of life			
(i) AD causes considerable psychological anxiety and results in a dramatic impact on the quality of life for both patients and their families	91.2		
(ii) The usefulness of PO-SCORAD for the self-assessment of AD in children suggests the importance of integrating the perspectives of the physician and the patient in the management of AD	91.2		

AD: atopic dermatitis; SCORAD: scoring atopic dermatitis; EASI: eczema area and severity index; PO-SCORAD: patient-oriented SCORAD. ^*∗*^43.8% neither agree nor disagree.

**Table 4:** Results regarding treatment and follow-up of patients with AD.

Item	Consensus (%)	Majority (%)	Discrepancy (%)
Substances that should be avoided			
(i) When the prick test is positive for any allergen with suspicion of clinical involvement, avoidance of these allergens as far as possible may be a useful complementary measure	83.8		
(ii) Patients with moderate-to-severe AD should follow a diet that does not include foods testing positive in the prick test or prick-prick test and that are clinically relevant for the patient	91.4		

Topical and anti-inflammatory treatment			
(i) The use of wet wraps increases the effect of topical corticosteroids	90.4		
(ii) Proactive “treatment”, for example, application for two times per week in long-term follow-up, can help reduce new flares	85.6		
(iii) Proactive “treatment” with application of tacrolimus ointment two times per week can help reduce new flares	91.2		
(iv) Simultaneous combination on the same location of topical glucocorticoids and topical calcineurin inhibitors does not seem to be useful		72.4	
(v) Based on results of clinical trials of crisaborole, this is not the treatment of choice for severe AD	92.4		

Antipruritic treatment			
(i) There are no sufficient bibliographic references supporting the general use of first- and second-generation antihistamines for treating pruritus in AD	85.7		
(ii) First- and second-generation antihistamines, in general, are not useful for systemic treatment of AD			64.8

Allergen-specific immunotherapy (allergen-SIT)			
(i) Allergen-SIT has positive effects in some sensitized patients with AD		76.2	
(ii) AD is not a contraindication for the use of immunotherapy in patients with allergic respiratory diseases (allergic rhinoconjunctivitis, allergic bronchial asthma)	92.4		

Systemic treatments			
(i) With the current immune response modifiers, the therapeutic needs of patients with severe AD are not sufficiently covered	97.6		
(ii) In the treatment of severe AD, cyclosporine has an adequate risk-benefit ratio	80.0		
(iii)With phototherapy, the therapeutic needs of patients with severe AD are not sufficiently covered	92.8		
(iv) In the treatment of severe AD, phototherapy has an adequate risk-benefit ratio			69.5

New systemic treatments			
(i) Treatment with biologic drugs should be considered in patients with severe AD not controlled with conventional systemic and topical treatment	93.6		
(ii) The objectives of these new biologic drugs should be targeting mainly cytokines involved in Th2 allergic inflammation such as IL-4, IL-5, IL-13, and IL-31	92.0		
(iii) Dupilumab has the potential to become the new first-line reference treatment for patients with moderate-to-severe AD who are candidates for systemic treatment (with or without topical treatment)	90.4		
(iv) According to results of phase II studies, JAK inhibitors will be a future treatment of AD			60.0^*∗*^

AD: atopic dermatitis; SIT: specific immunotherapy; IL: interleukin; JAK : Janus kinase. ^*∗*^60.0% neither agree nor disagree.

## Data Availability

The data used to support the findings of this study are included within the article.

## Supplementary Materials

Supplementary Materials Study questionnaire. Section 1: definition and diagnosis. Section 2: differential diagnosis. Section 3: severity of AD. Section 4: etiology and physiopathology. Section 5: comorbidities of AD. Section 6: health-related quality of life. Section 7: treatment and follow-up.
