# Supplementary material for: Consensus on the Clinical Approach to Moderate-to-Severe Atopic Dermatitis in Spain: A Delphi Survey
Source: Dermatol Res Pract. 2020 Apr 7;2020:1524293. doi: 10.1155/2020/1524293 (PMC7166259; doi:10.1155/2020/1524293)
Supplement: Supplementary Materials — Study questionnaire. Section 1: definition and diagnosis. Section 2: differential diagnosis. Section 3: severity of AD. Section 4: etiology and physiopathology. Section 5: comorbidities of AD. Section 6: health-related quality of life. Section 7: treatment and follow-up. [file 1524293.f1.pdf]

Study questionnaire

Section 1: Definition and diagnosis (14 items)

Item	Level of agreement
– AD can also develop *de novo* in adults or young adults, or even in advanced age	□ 1 □ 2 □ 3 □ 4 □ 5
– AD is a multifaceted disease that is derived from the interactions of multiple factors, including the skin components (cellular and extracellular components that form the skin barrier)	□ 1 □ 2 □ 3 □ 4 □ 5
– AD is a multifaceted disease that is derived from the interactions of multiple factors, including the immune system (innate and adaptative)	□ 1 □ 2 □ 3 □ 4 □ 5
– AD is a multifaceted disease that is derived from the interactions of multiple factors, including the skin microbiome	□ 1 □ 2 □ 3 □ 4 □ 5
– AD is a multifaceted disease that is derived from the interactions of multiple factors, including genetic factors	□ 1 □ 2 □ 3 □ 4 □ 5
– AD is a multifaceted disease that is derived from the interactions of multiple factors, including environmental factors	□ 1 □ 2 □ 3 □ 4 □ 5
– Currently, the diagnosis and assessment of the severity of AD are made on clinical grounds	□ 1 □ 2 □ 3 □ 4 □ 5
– The clinical criteria defined by Hanifin and Rajka are used for the clinical diagnosis of AD	□ 1 □ 2 □ 3 □ 4 □ 5
– The clinical presentation of AD depends on the age of the patients	□ 1 □ 2 □ 3 □ 4 □ 5
– With greater frequency, children < 2 years of age and adults present involvement of the face and neck, in addition, adults also present involvement of the flexor and extensor surfaces	□ 1 □ 2 □ 3 □ 4 □ 5
– Some forms of presentation seen in adults include, for example, dermatitis of the head and neck, chronic eczema of the hands and multiple zones of lichenification or prurigo	□ 1 □ 2 □ 3 □ 4 □ 5
– The classification of “intrinsic” AD (not associated with IgE) and “extrinsic” AD (associated with IgE) has practical implications related to specific avoidance strategies in the management of the disease	□ 1 □ 2 □ 3 □ 4 □ 5
– The blood eosinophil count is not a useful biomarker in AD	□ 1 □ 2 □ 3 □ 4 □ 5
– Currently there are no validated biomarkers that help in the diagnosis of AD	□ 1 □ 2 □ 3 □ 4 □ 5

AD: atopic dermatitis, IgE: immunoglobulin E

Section 2: Differential diagnosis (1 item)

Item	Level of agreement
– In certain situations, skin biopsy should be considered to exclude other conditions, such as early stage T-cell cutaneous lymphoma, psoriasis or dermatitis herpetiformis, among others	□ 1 □ 2 □ 3 □ 4 □ 5

Section 3: Severity of AD (2 items)

Item	Level of agreement
– The SCORAD index is used to quantify the severity of the disease in order to assess the comparative efficacy of treatments and progression of the disease in routine clinical practice	□ 1 □ 2 □ 3 □ 4 □ 5
– The EASI score is a validated scale that is not used in routine clinical practice	□ 1 □ 2 □ 3 □ 4 □ 5

AD: atopic dermatitis; SCORAD: scoring atopic dermatitis; EASI: eczema area and severity index.

Section 4: Etiology and physiopathology (4 items)

Item	Level of agreement
– A high percentage of patients with AD also present food allergy, allergic rhinitis or asthma	□ 1 □ 2 □ 3 □ 4 □ 5
– Treatments oriented to increase filaggrin expression are useful in the management of AD in a particular group of patients	□ 1 □ 2 □ 3 □ 4 □ 5
– Currently there is an unmet need in the treatment of moderate-severe AD	□ 1 □ 2 □ 3 □ 4 □ 5
– Biologic drugs are especially promising for adult patients with moderate or severe forms of the disease	□ 1 □ 2 □ 3 □ 4 □ 5

AD: atopic dermatitis.

Section 5: Comorbidities of AD (4 items)

Item	Level of agreement
– AD in adults is frequently associated with allergic comorbidities	□ 1 □ 2 □ 3 □ 4 □ 5
– AD in children is frequently associated with allergic comorbidities	□ 1 □ 2 □ 3 □ 4 □ 5
– The relative risk of suffering immune-mediated inflammatory diseases such as rheumatoid arthritis and chronic inflammatory bowel disease is higher in patients with AD than in the general population	□ 1 □ 2 □ 3 □ 4 □ 5
– Children with AD are more prone to suffer from mental disorders such as depression, anxiety, behavior disorders, among others	□ 1 □ 2 □ 3 □ 4 □ 5

AD: atopic dermatitis.

Section 6: Health-related quality of life (2 items)

Item	Level of agreement
– AD causes considerable psychological anxiety and results in a dramatic impact on the quality of life both for patients and their families	□ 1 □ 2 □ 3 □ 4 □ 5
– The usefulness of PO-SCORAD for the self-assessment of AD in children suggests the importance of integrating the perspectives of the physician and the patient in the management of AD	□ 1 □ 2 □ 3 □ 4 □ 5

AD: atopic dermatitis; PO-SCORAD: patient-oriented SCORAD.

Section 7: Treatment and follow-up (19 items)

Item	Level of agreement
Substances that should be avoided	
– When the prick test is positive for any allergen with suspicion of clinical involvement, avoidance of these allergens as far as possible may be a useful complementary measure	□ 1 □ 2 □ 3 □ 4 □ 5
– Patients with moderate-to-severe AD should follow a diet that does not include foods testing positive in the prick test or prick-prick test and that are clinically relevant for the patient	□ 1 □ 2 □ 3 □ 4 □ 5
Topical anti-inflammatory treatment	
– The use of wet wraps increases the effect of topical corticosteroids	□ 1 □ 2 □ 3 □ 4 □ 5
– Proactive "treatment", for example, application two times per week in long-term follow-up, can help reduce new flares	□ 1 □ 2 □ 3 □ 4 □ 5
– Proactive "treatment" with application of tacrolimus ointment two times per week can help reduce new flares	□ 1 □ 2 □ 3 □ 4 □ 5
– Simultaneous combination on the same location of topical glucocorticoids and topical calcineurin inhibitors does not seem to be useful	□ 1 □ 2 □ 3 □ 4 □ 5
– Based on results of clinical trials of crisaborole, this is not the treatment of choice for severe AD	□ 1 □ 2 □ 3 □ 4 □ 5
Antipruritic treatment	
– There are no sufficient bibliographic references supporting the general use of first- and second-generation antihistamines for treating pruritus in AD	□ 1 □ 2 □ 3 □ 4 □ 5
– First- and second-generation antihistamines, in general, are not useful for	□ 1 □ 2 □ 3 □ 4 □ 5

systemic treatment of AD	
Allergen-specific immunotherapy (allergen-SIT)	
– Allergen-SIT has positive effects in some sensitized patients with AD	□ 1 □ 2 □ 3 □ 4 □ 5
– AD is not a contraindication for the use of immunotherapy in patients with allergic respiratory diseases (allergic rhinoconjunctivitis, allergic bronchial asthma)	□ 1 □ 2 □ 3 □ 4 □ 5
Systemic treatments	
– With the current immune response modifiers, the therapeutic needs of patients with severe AD are not sufficiently covered	□ 1 □ 2 □ 3 □ 4 □ 5
– In the treatment of severe AD, cyclosporine has an adequate risk-benefit ratio	□ 1 □ 2 □ 3 □ 4 □ 5
– With phototherapy, the therapeutic needs of patients with severe AD are not sufficiently covered	□ 1 □ 2 □ 3 □ 4 □ 5
– In the treatment of severe AD, phototherapy has an adequate risk-benefit ratio	□ 1 □ 2 □ 3 □ 4 □ 5
New systemic treatments	
– Treatment with biologics should be considered in patients with severe AD not controlled with conventional systemic and topical treatment	□ 1 □ 2 □ 3 □ 4 □ 5
– The objectives of these new biologics should be targeting mainly cytokines involved in Th2 allergic inflammation such as IL-4, IL-5, IL-13, and IL-31	□ 1 □ 2 □ 3 □ 4 □ 5
– Dupilumab has the potential to become the new first-line reference treatment for patients with moderate-to-severe AD who are candidates to systemic treatment (with or without topical treatment)	□ 1 □ 2 □ 3 □ 4 □ 5
– According to results of phase II studies, JAK inhibitors will be a future treatment of AD	□ 1 □ 2 □ 3 □ 4 □ 5

AD: atopic dermatitis; SIT: specific immunotherapy; IL: interleukin; JAK: Janus kinase.
